# Association and Discrimination of Urinary System Stone Disease Using Anthropometric Indices, Lifestyle Behaviors, and Artificial Intelligence: A Multicenter Case–Control Study

**DOI:** 10.3390/diagnostics16213557

**Published:** 2026-10-28

**Authors:** Mehmet Erhan Aydın, Coşkun Kaya, Aykut Aykaç, Kadir Yildirim, Mucahit Karaduman, Fatih Bicaklioglu, Yiğit Kudret Akyol, Bayram Kolcu, Mehmet Ezer, Ferhat Yakup Suceken, Hikmet Yasar, Muhammed Yildirim, Kemal Sarica

**Affiliations:** 1 Department of Urology, Eskişehir City Health Application and Research Center, University of Health Sciences, 26080 Eskişehir, Turkey; 2 Department of Urology, Elazığ Fethi Sekin City Hospital, University of Health Sciences, 23280 Elazığ, Turkey; 3 Department of Software Engineering, Malatya Turgut Özal University, 44100 Malatya, Turkey; 4 Department of Urology, Kartal Dr. Lütfi Kırdar City Hospital, 34865 Istanbul, Turkey; 5 Department of Urology, Kafkas University Medical School, 36040 Kars, Turkey; 6 Department of Urology, Memorial Göztepe Hospital, 34634 Istanbul, Turkey; 7 Department of Urology, Sancaktepe Şehit Prof. Dr. İlhan Varank Training and Research Hospital, 34785 Istanbul, Turkey; 8 Department of Artificial Intelligence and Data Engineering, Fırat University, 23119 Elazığ, Turkey; 9 Department of Urology, Hamidiye Faculty of Medicine, University of Health Sciences, 34668 Istanbul, Turkey; 10 Department of Urology, Faculty of Medicine, Biruni University, 34015 Istanbul, Turkey

**Keywords:** anthropometry, machine learning, urolithiasis, body mass index, lifestyle

## Abstract

**Background/Objectives:** Urinary system stone disease is common and highly recurrent, and body mass index (BMI) poorly reflects the central adiposity linked to stone risk. We compared BMI with five next-generation anthropometric indices (A Body Shape Index [ABSI], Body Roundness Index [BRI], Waist-to-Height Ratio [WtHR], Conicity Index [ConI], Waist-to-Hip Ratio [WHR]) for their association with stone disease, and developed a hybrid autoencoder-based artificial intelligence model integrating anthropometric, clinical, and lifestyle data. **Methods:** This multicenter case–control study included 1195 participants (562 stone cases, 633 controls) from six centers in Turkey. Thirty-one variables were grouped into three subgroups (basic measurements, derived indices, clinical/lifestyle data), each processed by a separate autoencoder; combined features were classified with XGBoost, Cubic SVM, Fine KNN, and logistic regression. Each index’s independent association was assessed by quartile-based logistic regression, and discriminative power by ROC/AUC. **Results:** BMI showed no between-group difference (*p* = 0.919) or discriminative power (AUC = 0.498). ABSI, BRI, WtHR, ConI, and WHR were all significantly higher in the stone group, showed positive trends across quartiles after full adjustment (all p-trend ≤ 0.001), and outperformed BMI (AUC 0.547–0.564); associations were strongest for ConI (aOR = 2.62) and ABSI (aOR = 2.57). The hybrid AI model performed best with XGBoost (77% accuracy), followed by Fine KNN (71%), logistic regression (69%), and Cubic SVM (68%). **Conclusions:** Unlike BMI, next-generation indices reflecting central adiposity are independently and consistently associated with stone disease, and a multivariable AI model combining anthropometric, clinical, and lifestyle data reached 77% accuracy in separating stone patients from controls on a single test split. These findings are a hypothesis-generating risk-stratification signal that requires external validation, and they do not support clinical screening.

## 1. Introduction

Urinary system stone disease is one of the most common urological conditions worldwide, with its prevalence varying markedly by region: 7–13% in North America, 5–9% in Europe, and 1–5% in Asia [1]. According to the Global Burden of Disease (GBD) 2021 study, 106 million new cases of urolithiasis were recorded worldwide in 2021, reflecting a 26.7% increase compared with 2000 [2]; in the United States, National Health and Nutrition Examination Survey (NHANES) data indicate a prevalence of 10.9% in men and 9.5% in women [3]. A multicenter epidemiological study conducted in Turkey reported a lifetime prevalence of stone disease of 11.1% and an annual incidence of 1.7% [4]. Stone disease is further notable for its high recurrence rate (50–72%) [5] and its association with the increasing prevalence of metabolic conditions such as obesity and diabetes [1].

Obesity and weight gain have long been recognized as classical risk factors for urinary system stone disease. In two large prospective cohort studies, the risk of stone formation was significantly higher in men weighing more than 100 kg compared with those weighing less than 68.2 kg, and marked weight gain since early adulthood was similarly associated with increased risk [1]. Stone disease has also been linked to central (abdominal) obesity: the presence of four or more features of the metabolic syndrome nearly doubled the odds of self-reported stone disease [1]. However, body mass index (BMI) is insensitive to regional fat distribution and cannot distinguish central adiposity [6]. This limitation has driven the development of alternative anthropometric indices sensitive to central adiposity: five next-generation indices sensitive to central adiposity have been described: A Body Shape Index (ABSI), Body Roundness Index (BRI), Waist-to-Height Ratio (WtHR), Conicity Index (ConI), and Waist-to-Hip Ratio (WHR). The ConI models waist circumference relative to body weight and height [6]. ABSI renders waist circumference independent of BMI and height, thereby predicting mortality risk independently of BMI [7]. The BRI, derived from a geometric model based on waist circumference and height, offers a modest improvement over BMI in predicting total and visceral body fat [8].

The association between these next-generation anthropometric indices and urinary system stone disease has largely been examined in single-country cross-sectional datasets such as NHANES. In a cohort of 121,605 participants from the Taiwan Biobank, BRI and WtHR (together with BMI and WHR) were shown to be significantly associated with stone disease; however, in the fully adjusted longitudinal analysis from the same study, ConI did not show this association [9]. Central adiposity measured by the WHR has been shown to increase stone disease risk independently of BMI at a causal level in a UK Biobank-based Mendelian randomization study [10]. These findings have been consistent across different populations [5,11,12,13,14,15,16,17].

Although the use of artificial intelligence (AI) in urology is increasingly widespread in areas such as diagnosis, treatment success, and recurrence prediction [18,19], a comprehensive systematic review evaluating AI applications in urolithiasis found that only four of 71 identified articles focused on epidemiology and risk factor prediction, and that these four relied on invasive data types (clinical/biochemical parameters, genetic polymorphisms) [20]. The closest example based purely on anthropometric and lifestyle data is a model developed from 976 Chilean participants that predicted stone risk with 88% accuracy [21]; other AI-based stone prediction studies rely predominantly on laboratory or urinary data [22,23,24,25,26].

Previous studies have linked BRI, ABSI, ConI, WtHR, and WHR to stone disease, mostly in cohorts from the United States, Taiwan, and China [5,9,11,12,13,14,15,16,17]. Some compared several indices in one sample [9,13], and stone status was self-reported in the Taiwan Biobank and NHANES studies [5,9,11]. Machine learning has been applied to the ZJU composite metabolic index in NHANES data [27], to composite obesity indices in diabetic-hypertensive adults [28], and to survey data from a Chilean general-population sample [21]. We found no earlier study that evaluated BMI, ABSI, BRI, WtHR, ConI, and WHR together in a multicenter case–control sample with stone status confirmed by imaging, surgical history, and clinical history, and that analyzed the same sample with logistic regression, ROC analysis, and an autoencoder-based classification pipeline. The present study addresses this gap.

We hypothesized that ABSI, BRI, WtHR, ConI, and WHR would show a stronger and more consistent independent association with urinary system stone disease than BMI, and that a hybrid autoencoder-based AI model integrating anthropometric, clinical, and lifestyle data would reach higher classification accuracy than any single anthropometric index alone. The aims were, first, to evaluate the association of BMI and five next-generation anthropometric indices (ABSI, BRI, WtHR, ConI, WHR) with primary urinary system stone disease by multivariable logistic regression and receiver operating characteristic (ROC) curve and area under the curve (AUC) comparison, and second, to develop a hybrid autoencoder-based AI model integrating anthropometric measurements, derived indices, and clinical and lifestyle variables and to compare the performance of four classifiers (XGBoost, Cubic SVM, Fine KNN, logistic regression).

## 2. Materials and Methods

### 2.1. Study Design and Setting

This study is a multicenter case–control study conducted at six tertiary care centers in Turkey between January 2025 and June 2025. The study was conducted in accordance with the principles of the Declaration of Helsinki and was approved by the Scientific Research Ethics Committee of Eskişehir City Hospital (Approval No: ESH/BAEK 2025/93, Approval Date: 7 January 2025); written informed consent was obtained from all participants prior to enrollment. Completed STROBE (Table S3) and TRIPOD+AI (Table S4) checklists are provided in the Supplementary Materials.

### 2.2. Participants

A total of 1195 participants were included in the study: 562 patients with a confirmed diagnosis of urinary system stone disease (case group) and 633 control participants without stone disease, recruited from the same six centers. The presence or absence of stone disease was confirmed through a combined assessment of imaging findings, surgical history, and clinical history. Control group participants were randomly recruited from the same centers throughout the study period. Individuals with a single kidney or chronic kidney disease were excluded from both groups.

The 562 patients in the case group are identical to the cohort previously analyzed by our team to investigate the risk of stone recurrence [29]. In the present study, this cohort was combined with a newly collected, stone-free control group to address a different research question: rather than recurrence risk, the distinction between individuals with and without stone disease. No outcome data related to recurrence from the previous study were used in this analysis.

### 2.3. Data Collection

Data were collected across all six participating centers using a structured case report form (CRF), applied in a standardized manner by the investigators. The form included 25 variables covering each participant’s demographic characteristics, anthropometric measurements, comorbidities, and lifestyle behaviors: age, sex, education level, diabetes, hypertension, cardiovascular disease, inflammatory bowel disease, history of bowel surgery, family history of stone disease, daily water consumption, daily physical activity level, tea consumption, coffee consumption, salt intake, animal protein intake, climate type, occupation type, medication associated with stone formation, sweating, smoking, alcohol, height, weight, waist circumference, and hip circumference. Six derived anthropometric indices (Section 2.4) were added to these 25 variables, giving 31 variables in total (5 basic measurements, 6 indices, and 20 clinical, demographic, and lifestyle variables; Section 2.5). The dataset contained no missing values for any of the 31 variables across the 1195 participants; therefore, no imputation procedure was required.

### 2.4. Anthropometric Measurements and Derived Indices

Waist circumference was measured at the level of the upper border of the iliac crest, and hip circumference at the widest (gluteal) point of the hips, using a non-stretchable measuring tape [7,8]. All anthropometric measurements were performed by experienced, trained nurses at each center. From these measurements, six anthropometric indices were calculated: BMI, ABSI [7], BRI [8], WtHR, ConI [6], and WHR. The formulas used for each index are presented in Table 1.

### 2.5. Variable Grouping for the Artificial Intelligence Model

Following data collection, the 6 derived indices calculated above were added to the 25 raw variables, yielding a total of 31 variables, which were divided into three conceptually distinct subgroups for the hybrid autoencoder-based classification framework: Part A consists of the basic anthropometric measurements (age, height, weight, waist circumference, hip circumference); Part B consists of the six derived anthropometric indices (BMI, ABSI, BRI, WtHR, ConI, WHR); and Part C consists of the remaining 20 clinical, demographic, and lifestyle variables described above (for the complete list of the 31 variables, their definitions, and coding scheme, see Table S1).

### 2.6. Artificial Intelligence Model Development

Consistent with the approach previously described by our team [29], a hybrid deep learning–machine learning framework was developed. The dataset was first randomly split into 80% training and 20% test subsets, with a fixed random state used to ensure reproducibility. The resulting test set contained 106 stone and 133 stone-free participants (44.4% and 55.6%), compared with 47.0% and 53.0% in the full sample. Each of the three variable subgroups (Part A, B, and C) was fed independently into a separate autoencoder architecture [30], consisting of an input layer followed by an encoding hidden layer, in order to obtain low-dimensional, information-rich feature representations; the Rectified Linear Unit (ReLU) [31] was used as the activation function. Each autoencoder was trained exclusively on the training subset; the test subset was withheld during this step and was only transformed using the already-fitted autoencoder weights, thereby preventing information leakage from the test data into the feature-extraction process. The resulting encoded representations were concatenated to form a single feature vector, which was then used to train and evaluate four classification algorithms: Extreme Gradient Boosting (XGBoost) [32], Cubic Support Vector Machine (Cubic SVM) [33], Fine K-Nearest Neighbor (Fine KNN) [34], and Logistic Regression [35]. In this study, individuals with stone disease were defined as the positive class (stone present), and individuals without stone disease as the negative class (stone absent). True positive (TP) refers to the number of correctly classified stone-positive cases; true negative (TN), the number of correctly classified stone-negative cases; false positive (FP), the number of cases incorrectly classified as stone-positive; and false negative (FN), the number of cases incorrectly classified as stone-negative. Classifier performance was evaluated using accuracy, sensitivity (recall), specificity, positive predictive value (PPV; precision), negative predictive value (NPV), and F1-score for the positive class. We also report the macro-averaged precision, recall, and F1-score, which are the unweighted means of the values for the stone-present and stone-absent classes, together with the Matthews correlation coefficient (MCC) and Cohen’s kappa. Performance metrics were calculated using the following formulas:

Accuracy = (TP + TN)/(TP + TN + FP + FN)(1)

Precision = TP/(TP + FP)(2)

Recall = TP/(TP + FN)(3)

F1-Score = 2 × (Precision × Recall)/(Precision + Recall)(4)

Specificity = TN/(TN + FP)(5)

Negative predictive value = TN/(TN + FN)(6)

MCC = (TP × TN − FP × FN)/√[(TP + FP)(TP + FN)(TN + FP)(TN + FN)](7)

Cohen’s kappa = (p_0_ − p_e_)/(1 − p_e_) (8)

Here p_0_ is the observed agreement, which equals accuracy, and p_e_ is the agreement expected by chance.

Accuracy reflects the proportion of correct predictions among all predictions and measures the overall success of the model. Precision indicates the proportion of truly positive cases among those predicted as positive, and is of value in settings where the risk of false positives leading to unnecessary further investigation (imaging, additional testing) is a concern. Recall, in turn, indicates how many of the true stone-positive cases were correctly identified; this is particularly important in the context of the present study, as avoiding the failure to identify individuals with stone disease (i.e., minimizing false negatives) is clinically critical. As the harmonic mean of precision and recall, the F1-score provides a balance between the two metrics in settings where the class distribution is not perfectly even (in this study, 562 stone-positive versus 633 stone-negative cases). Specificity and negative predictive value show how well stone-free individuals are identified.

The hyperparameters used for the autoencoder and classification models are presented in Table 2. These parameters define the model configurations employed during feature extraction and classification.

### 2.7. Statistical Analysis

The normality of the distribution of continuous variables was assessed using the Shapiro–Wilk test; normally distributed variables are presented as mean ± standard deviation (SD), and non-normally distributed variables as median (interquartile range, IQR). Categorical variables are presented as number (percentage). Differences between the groups with and without stone disease were assessed using the independent samples *t*-test for normally distributed continuous variables, the Mann–Whitney U test for non-normally distributed continuous variables, and the chi-square test for categorical variables.

Multivariable logistic regression analysis was performed to determine the independent association of each anthropometric index (BMI, ABSI, BRI, WtHR, ConI, WHR) with urinary system stone disease. In this analysis, each index was divided into quartiles (Q1–Q4) based on its distribution across the entire sample (*n* = 1195), with the lowest quartile (Q1) used as the reference category. Model 1 was adjusted for age and sex; Model 2 (the fully adjusted model) was additionally adjusted for education level, hypertension, diabetes, cardiovascular disease, smoking, alcohol use, family history of stone disease, daily water consumption, daily physical activity level, climate type, and medication use associated with stone formation. Because no linear relationship was assumed among their categories, education level, daily physical activity level, and climate type were included in the model as categorical (dummy) variables. Results are expressed as adjusted odds ratios (aOR) and 95% confidence intervals (CI). To assess the dose–response relationship across quartiles, a linear trend test was performed by entering the quartile rank of each index into the model as a continuous variable, and the resulting *p*-value was reported as “*p* for trend.” As a sensitivity analysis, Model 2 was additionally re-estimated after incorporating four covariates that showed significant univariate differences between groups in Table 3 but were not included in the primary Model 2 specification (history of bowel surgery, animal protein intake, coffee consumption, and sweating), to assess the robustness of the primary findings to this expanded adjustment set (Table S2).

Receiver operating characteristic (ROC) curve analysis (area under the curve, AUC) was performed to evaluate the discriminative power of BMI for urinary system stone disease in comparison with the next-generation anthropometric indices (ABSI, BRI, WtHR, ConI, WHR); 95% confidence intervals for the AUC values were calculated using the Hanley–McNeil method [36]. For every index, higher values were taken to indicate stone disease (stone present as the positive class). An AUC below 0.5 therefore means that cases tend to have lower values than controls, and an AUC whose 95% CI includes 0.5 cannot be distinguished from chance.

Statistical analyses were performed using the Python programming language (version 3.12; Python Software Foundation, Wilmington, DE, USA) with the SciPy (1.17), statsmodels (0.15), and scikit-learn (1.8) libraries; a two-tailed *p* < 0.05 was considered statistically significant.

## 3. Results

**Figure 1:**
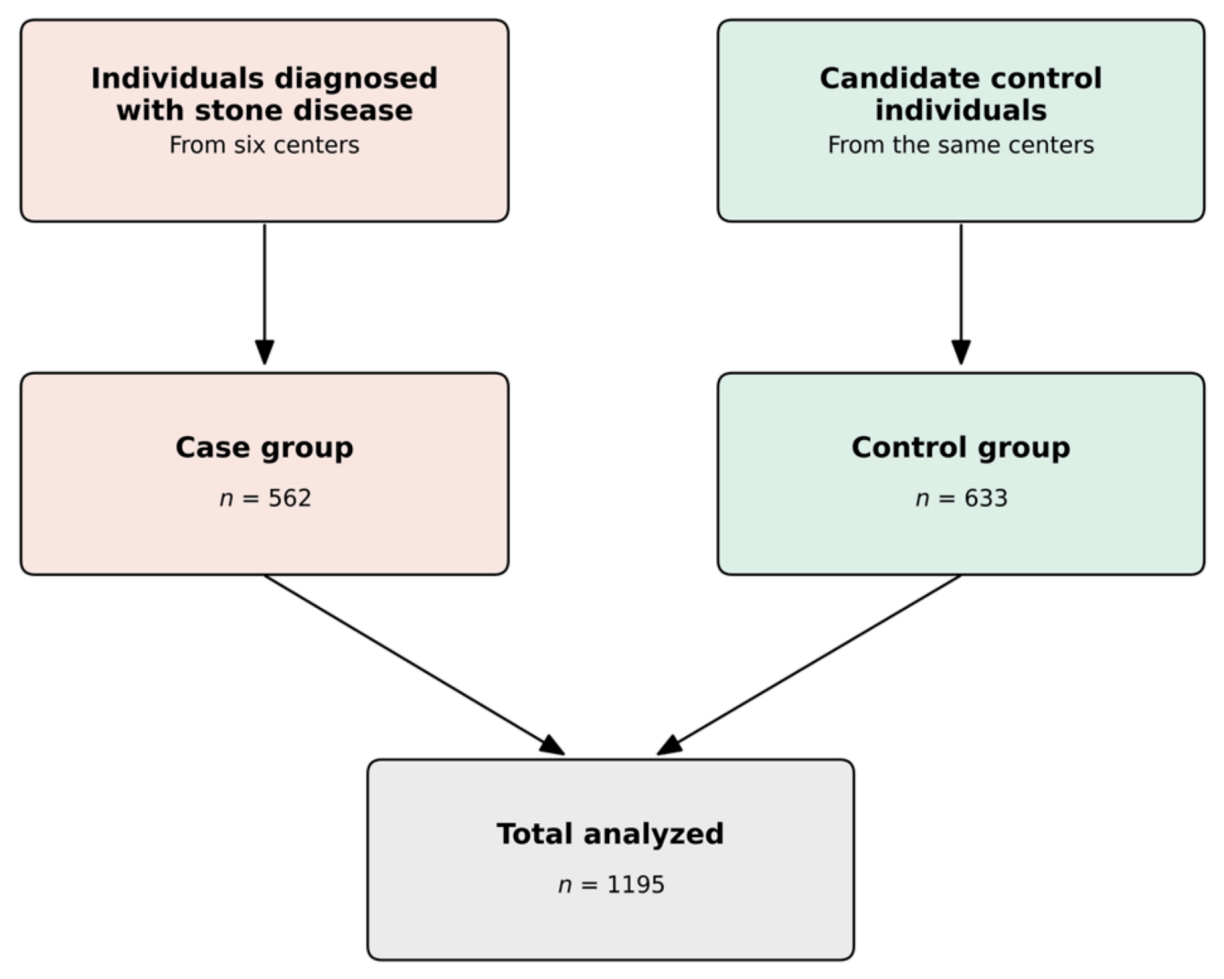
Participant flow diagram. The case group with a diagnosis of urinary system stone disease (*n* = 562) and the control group without stone disease (*n* = 633) were enrolled from six centers; individuals with a single kidney or chronic kidney disease were excluded from both groups, yielding a total of 1195 participants for analysis.

A total of 1195 participants (562 cases, 633 controls) were included in the study (Figure 1).

The demographic, clinical, and anthropometric characteristics of the participants are presented in Table 3. With regard to the basic anthropometric measurements, the stone-free group was significantly taller (*p* < 0.001) and heavier (*p* = 0.025) than the stone group, while the difference in age reached borderline significance (*p* = 0.047). No significant difference was observed between the groups in waist or hip circumference. Among the formulated anthropometric indices, ABSI, BRI, WtHR, ConI, and WHR were all significantly higher in the stone group (all *p* < 0.05), whereas no difference was observed between the groups with regard to BMI (*p* = 0.919). Among the clinical and lifestyle variables, hypertension (*p* = 0.001) and history of bowel surgery (*p* = 0.012) were significantly more common in the stone-free group, whereas family history of stone disease (*p* < 0.001), use of medication associated with stone formation (*p* < 0.001), sweating (*p* < 0.001), animal protein intake (*p* = 0.005), coffee consumption (*p* = 0.038), and climate type (*p* < 0.001) were significantly more common/higher in the stone group. No significant differences were observed between the groups with regard to sex, education level, diabetes, cardiovascular disease, inflammatory bowel disease, water consumption, daily physical activity, tea and salt intake, occupation type, smoking, or alcohol use.

The performance of the four classification algorithms was compared following autoencoder-based feature extraction (Table 4). The highest performance was achieved with the XGBoost classifier, with an accuracy of 77%, followed by Fine KNN (71%), logistic regression (69%), and Cubic SVM (68%), respectively. Precision, recall, and F1-score values reflected the same ranking, with XGBoost showing the most balanced and highest performance across all metrics. Based on these results, XGBoost was determined to be the most suitable classifier within the proposed hybrid autoencoder-classifier framework.

An examination of Table 4 reveals that the performance metrics achieved by the XGBoost classifier are superior to those of the other classifiers. XGBoost attained the highest classification performance, with an accuracy of 77.0% and a macro F1-score of 76.9%; furthermore, its MCC and Cohen’s Kappa values of 0.54 indicate a stronger classification agreement compared to the other models. XGBoost was followed by the Fine KNN (70.7%), Logistic Regression (69.0%), and Cubic SVM (68.2%) models. These results demonstrate that, among the models evaluated, XGBoost is the most successful approach for the classification of urinary system stone disease.

**Figure 2:**
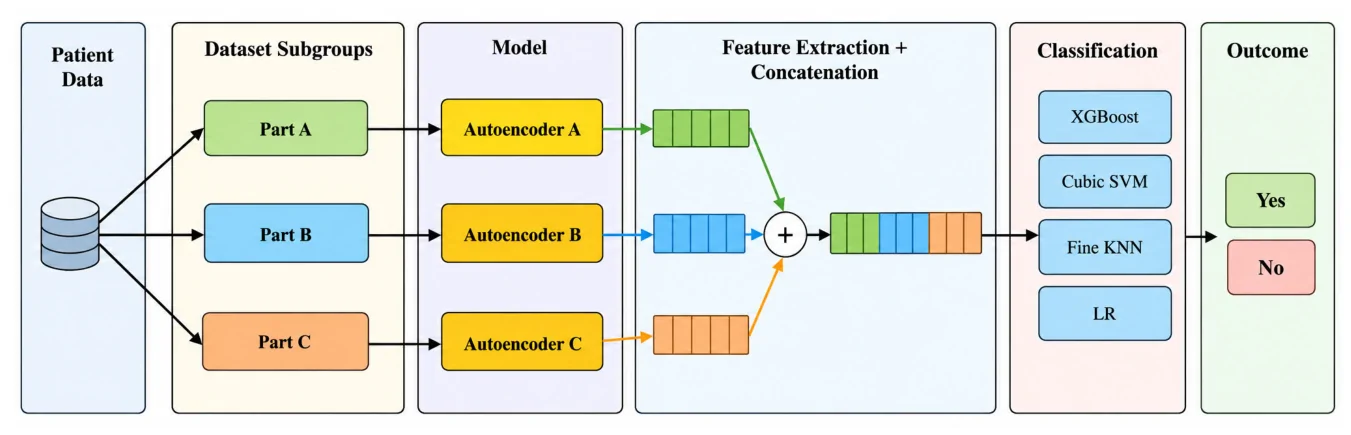
Overall structure of the developed hybrid autoencoder-based classification model. Patient data were divided into three conceptual subgroups (Part A: basic anthropometric measurements; Part B: derived anthropometric indices; Part C: clinical, demographic, and lifestyle variables), with each subgroup passed through a separate autoencoder architecture for feature extraction; the resulting feature vectors were concatenated, and stone disease status was classified using four classification algorithms (XGBoost, Cubic SVM, Fine KNN, and Logistic Regression (LR), as labeled in the figure). Green, blue, and orange denote Part A, Part B, and Part C and their encoded features, respectively; yellow boxes are the autoencoders. In the outcome box, “Yes” (green) denotes stone present and “No” (red) denotes stone absent.

The overall structure of the developed hybrid autoencoder-based classification model is shown in Figure 2.

**Figure 3:**
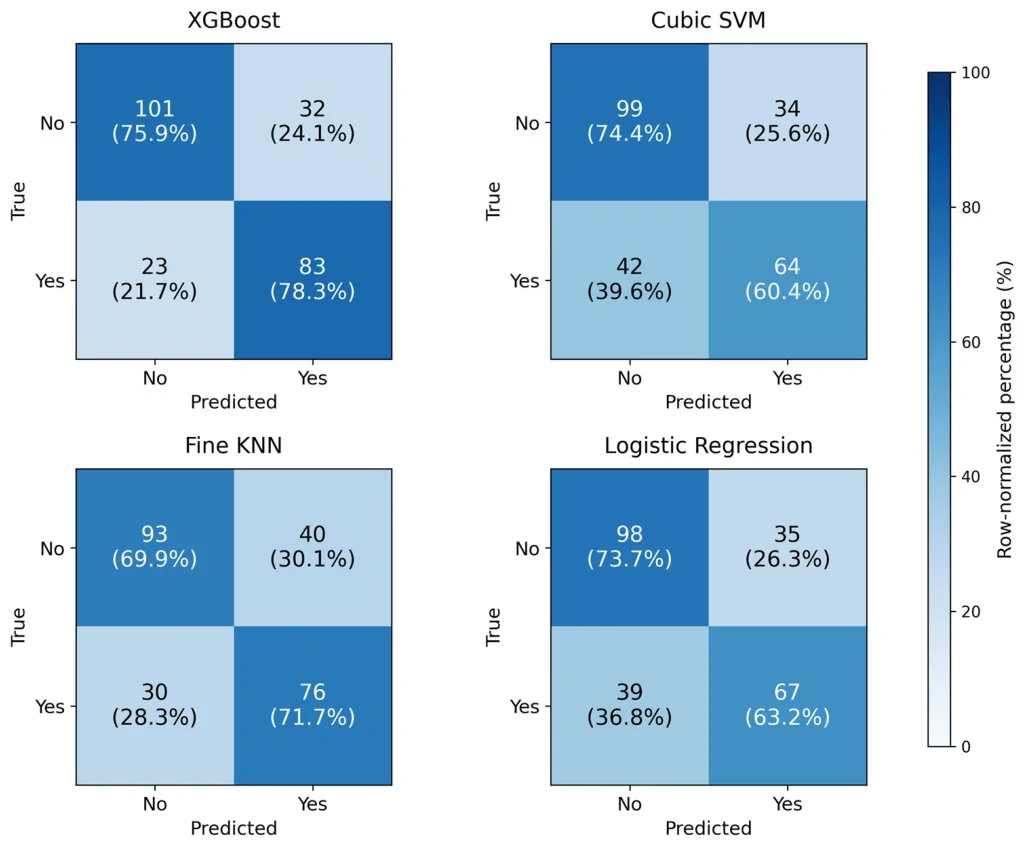
Confusion matrices for the classifiers (stone vs. no stone). Each cell shows the number of participants and the row-normalized percentage.

The confusion matrix for the classifiers is presented in Figure 3. For the 239 patients in the test set, XGBoost correctly classified 184 (accuracy = 77.0%), with a sensitivity of 78.3% (83/106), specificity of 75.9% (101/133), positive predictive value of 72.2% (83/115), and negative predictive value of 81.5% (101/124).

Among the classifiers used in the study, XGBoost yielded the most successful results. In the test set, which consisted of 239 patients (20% of the total *n* = 1195), XGBoost correctly classified 101 patients as not having stone disease and 83 patients as having stone disease; 32 patients were incorrectly classified as having stone disease, while 23 patients were incorrectly classified as not having stone disease. The lowest performance was observed with the Cubic SVM classifier, which misclassified 76 out of the 239 test cases. Sensitivity for the stone-present class was 78.3% with XGBoost, 71.7% with Fine KNN, 63.2% with logistic regression, and 60.4% with Cubic SVM, and specificity was 75.9%, 69.9%, 73.7%, and 74.4%, respectively (Table 4).

The association of each of the six anthropometric indices (BMI, ABSI, BRI, WtHR, ConI, WHR) with urinary system stone disease was evaluated using multivariable logistic regression, with each index divided into quartiles based on its distribution across the entire sample (Table 5). BMI showed no significant association in any quartile in either Model 1 or Model 2, and the linear trend was likewise non-significant (p-trend = 0.736 and 0.999, respectively). In contrast, ABSI, BRI, WtHR, ConI, and WHR all showed a significant positive trend across quartiles in the fully adjusted model (Model 2) (all p-trend values ≤0.001). The strongest association was observed for ConI: individuals in the highest quartile (Q4) had 2.62-fold higher odds of stone disease compared with those in the lowest quartile in Model 2 (95% CI: 1.80–3.81). A similarly strong association was observed for ABSI (Q4 aOR = 2.57, 95% CI: 1.78–3.70). Because BRI and WtHR are monotonic transformations of one another by formulation (Spearman ρ = 1.000), they yielded identical results in the quartile-based analysis (Q4 aOR = 1.90, 95% CI: 1.32–2.74); this is not a calculation error but a natural consequence of the mathematical relationship between the two indices. WHR also showed a significant dose–response relationship (Q4 aOR = 1.95, 95% CI: 1.32–2.87, p-trend < 0.001). In the sensitivity analysis incorporating history of bowel surgery, animal protein intake, coffee consumption, and sweating as additional covariates (Table S2), the overall pattern of associations remained unchanged: BMI showed no association in any quartile (p-trend = 0.827), while ABSI, BRI, WtHR, ConI, and WHR retained statistically significant positive trends across quartiles (all p-trend ≤ 0.003), with only minor attenuation in point estimates (e.g., ConI: Q4 aOR = 2.51 [95% CI: 1.71–3.68] vs. 2.57 in the primary model). These results indicate that the primary findings are robust to this expanded adjustment set. These findings indicate that next-generation anthropometric indices, by reflecting body fat distribution more accurately than BMI, show an independent and consistent association with urinary system stone disease.

**Figure 4:**
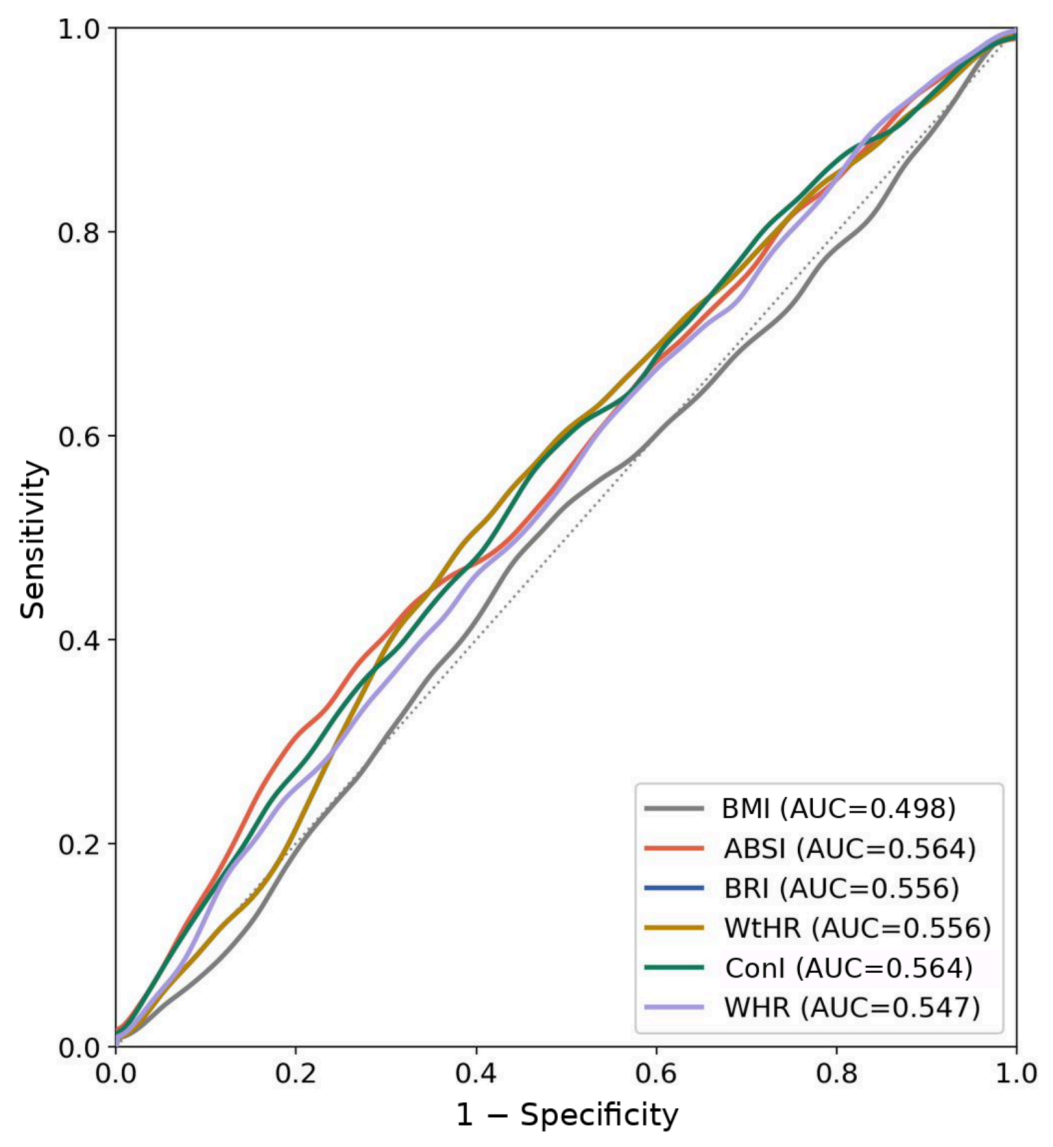
ROC curves showing the discriminative power of BMI and the next-generation anthropometric indices (ABSI, BRI, WtHR, ConI, WHR) for urinary system stone disease. For every index, higher values were taken to indicate stone disease (stone present as the positive class). The BMI curve tracked closest to the reference (random-chance) line, while the curves of the other five indices ran above BMI and close to one another. Because BRI and WtHR are monotonic transformations of one another by formulation, their curves overlap exactly.

The discriminative power of BMI for urinary system stone disease was found to be at chance level (AUC = 0.498, 95% CI: 0.465–0.531). With higher values indicating stone disease (Section 2.7), an AUC marginally below 0.5 means that BMI ranked slightly lower in cases than in controls, and the 95% CI includes 0.5. All five next-generation anthropometric indices showed higher and closely comparable AUC values than BMI: ABSI and ConI exhibited the highest discriminative power (both AUC = 0.564), followed by BRI and WtHR, which are mathematically monotonic transformations of one another (both AUC = 0.556), and finally WHR (AUC = 0.547) (Table 6, Figure 4). Examination of the ROC curves showed that the BMI curve tracked closest to the reference line, whereas the curves of the other five indices ran close to one another and above that of BMI; the curves for BRI and WtHR overlapped exactly, owing to their mathematical identity. Overall, although the discriminative power of all indices remained modest (AUC < 0.60), the findings support that next-generation indices carry a weak but consistent discriminative ability for stone disease.

## 4. Discussion

In this multicenter case–control study, a clear divergence was observed among the six anthropometric measures evaluated. The classical BMI showed no association with stone disease in the descriptive comparison, in the multivariable logistic regression analysis (p-trend = 0.736 and 0.999), or in the discriminative power assessment (AUC = 0.498, 95% CI: 0.465–0.531). In contrast, the five next-generation indices reflecting central adiposity (ABSI, BRI, WtHR, ConI, and WHR) showed an independent positive trend across quartiles in the fully adjusted model (all p-trend ≤ 0.001) and had higher discriminative power than BMI (AUC 0.547–0.564). The strongest associations were observed for ConI and ABSI (Q4 aOR of 2.62 and 2.57, respectively). BRI and WtHR yielded identical results owing to their mathematical identity (Spearman ρ = 1.000). These associations remained materially unchanged in a sensitivity analysis incorporating additional covariates (history of bowel surgery, animal protein intake, coffee consumption, and sweating; Table S2), supporting the robustness of these findings. Consistent with this pattern, the highest performance in the hybrid autoencoder-based model was likewise achieved with XGBoost (77% accuracy); however, as no SHapley Additive exPlanations (SHAP) analysis was performed, it was not possible to determine which variable group this performance was primarily attributable to. As shown in Table 3, although the control group was significantly taller and heavier than the patients with stone disease, there were no significant differences between the groups regarding waist and hip circumference measurements. Since anthropometric indices are derived from mathematical combinations of these primary measurements, differences in height and weight can lead to significant variations in the derived indices, even when waist and hip circumferences are similar. Consequently, the associations across quartiles observed in these indices may be influenced not only by actual differences in fat distribution but also by the mathematical structure of the ratios and the specific characteristics of the case–control sample. Therefore, the current findings should not be interpreted as direct evidence of an independent biological effect of body fat distribution on stone disease. Our findings suggest that, unlike BMI, these derived anthropometric indices capture differences in body proportions associated with stone disease, although these associations may partly reflect their mathematical structure rather than independent differences in fat distribution.

Our findings largely align with the current literature, although they diverge on certain points. ABSI and BRI have been consistently reported to show a stronger association with stone disease than BMI in NHANES-based studies [11,12,13]; higher AUC values than BMI have similarly been reported for discriminative power (AUC 0.589–0.597 versus 0.558–0.570 for BMI) [12,13]. In our cohort, however, the discriminative power of BMI was even lower than in these studies, not distinguishable from chance (AUC = 0.498; the 95% CI includes 0.5); this may reflect a difference between the population-based NHANES sample and our tertiary-care clinical population, although this interpretation remains speculative.

When the five significant indices were examined in relation to one another, the strong correlation between ConI and ABSI (Spearman ρ = 0.95; Figure S1) suggests that they carry overlapping information regarding central adiposity. The identical results for BRI and WtHR (see above) reflect a single relationship expressed through two formulas and are not two independent confirmations. A plausible biological basis for this pattern lies in the association of central/visceral adiposity with metabolic pathways that disrupt the urinary lithogenic profile (hyperuricosuria, hyperoxaluria, hyperinsulinemia-related hypercalciuria, hypocitraturia) [1,10]. Specifically for WHR, a UK Biobank cohort reported that each 1-SD increase in WHR increases stone risk by approximately 24%, independently of BMI, and that part of this effect (12–15%) is mediated through an increase in serum calcium [10]. This mechanism could not be directly tested in our data (see Limitations).

Our findings also show partial overlap with recent cohort studies conducted in different populations. In a study following 26,594 non-diabetic individuals in Beijing, only BRI retained significance after full adjustment, while BMI became non-significant [15]; this pattern shows a clear parallel with our BMI finding. The association of BRI and ConI with stone risk has also been confirmed in other large cohorts [5,14,17], and the positive association of WHR in a hypertensive population is directionally consistent with our finding as well [16]. On the other hand, in a longitudinal analysis from the Taiwan Biobank, BMI retained significance while ConI did not [9], a pattern that is the exact opposite of ours. This discrepancy may stem from differences in population structure, stone definition (self-report versus imaging), or follow-up duration, but it indicates that the literature has not yet reached consensus on this point.

The closest conceptually related precedent to our study is a cohort of 976 Chilean participants, conducted using survey data alone without laboratory or imaging data, which achieved 88% accuracy [21]. Possible reasons why our 77% result falls below this accuracy include differences in the variable sets used, differences in sampling method (anonymous self-report via social media), and the absence of cross-validation in our study. In contrast, studies using direct laboratory/urinary data report markedly higher performance: 99% accuracy with Random Forest and Extra Trees [23], 83% accuracy and 100% sensitivity with the KNN algorithm using urinary conductivity/pH/calcium data [24], and an AUC of 0.965–0.968 with combined urinary and clinical parameters [25,26]. The inclusion of invasive data carrying direct diagnostic information in these studies provides a significant performance advantage compared with our non-invasive design; our more limited performance should be viewed as a trade-off inherent to a low-cost, non-invasive design. One of the methodologically closest precedents is the ZJU (Zhejiang University) index study conducted using NHANES data (*n* = 11,317), in which a single composite metabolic index was evaluated using both logistic regression and ML models (best performance with XGBoost, AUC = 0.638) and identified as the most influential predictor through SHAP analysis [27]. However, the ZJU index is not fully non-invasive, as it includes laboratory parameters such as fasting glucose and triglycerides. Similarly, in a study comparing nine ML algorithms with SHAP in diabetic-hypertensive adults, the best performance was achieved with Random Forest (AUC = 0.895), with relative fat mass and lipid accumulation product identified as the strongest predictors [28]. The fact that both studies offer SHAP-based interpretability also highlights this limitation in our own model (see Limitations).

In this context, our study represents one of the non-invasive approaches directed toward the epidemiological risk assessment domain, which, as shown by Altunhan et al. [20], remains relatively understudied in the literature compared with the diagnosis and treatment domain that has predominantly been the focus of AI applications in urolithiasis. The global economic burden of stone disease is substantial; in the United States, annual treatment costs were reported to have exceeded 5 billion dollars in 2005, with an additional projected increase of 1.24 billion dollars by 2030 [37]. Although these indices can be calculated from routinely measured anthropometric parameters without additional cost, their limited discriminative performance (AUC < 0.60) does not support their use as standalone screening tools. Notably, despite the limited discriminative power of the individual indices, the autoencoder-based hybrid model achieved 77% accuracy by jointly using all variable groups; this suggests that a multivariable approach may be more informative than single indices, as a hypothesis-generating signal for risk stratification that requires external validation.

The cohort overlap of the case group with our previous study [29] is detailed in Section 2; no outcome data related to recurrence from the previous study were used in this analysis. In both studies, using a similar autoencoder-based framework, the highest performance was achieved with XGBoost (77% in the present study, 75.22% in the previous study); this similarity suggests that the approach used consistently identifies the best-performing classifier across different clinical questions.

Our study has several methodological strengths. The multicenter design, spanning six centers from different regions of Turkey, provides broader representativeness compared with single-center studies. The comparison of six indices within the same cohort and the same framework offers a more comprehensive evaluation than studies in the literature limited to one or two indices. Two advantages stand out with regard to data quality as well. Objective confirmation of the stone diagnosis through imaging and surgical history provides a more reliable outcome measure compared with studies relying on self-report (e.g., [9]). The collection of lifestyle data through a structured CRF may also have achieved a higher degree of standardization compared with anonymous self-report surveys [21]. Finally, the combined use of classical statistics and machine learning enabled our findings to be supported by two complementary approaches.

Two findings in the descriptive comparison (Table 3) appear, at first glance, to run counter to established expectations. First, the higher frequency of hypertension in the stone-free group aligns with the mixed association pattern reported in the literature [1] and with Mendelian randomization evidence indicating that hypertension has no direct causal relationship with stone formation [10]. As this variable was used in the models as an adjustment covariate rather than a primary variable of interest, no causal interpretation is made. Second, the higher frequency of history of bowel surgery in the stone-free group appears to run counter to the known surgical types (e.g., bariatric surgery, small bowel resection) associated with increased stone risk through enteric hyperoxaluria [38]. This unexpected direction may stem from the fact that the history of bowel surgery variable in our data collection form was defined without distinguishing surgical type (e.g., it may also include unrelated procedures such as appendectomy); statistical power may also have been limited by small cell counts. Both findings were observed only at the descriptive level and are unrelated to the primary hypothesis (anthropometric indices); they should therefore be interpreted with caution and evaluated in dedicated future studies.

### Limitations

This study has several limitations. First, causality cannot be established owing to the cross-sectional case–control design. Furthermore, the case–control design does not permit estimation of disease incidence or prospective predictive performance. The quartile cut-points were derived from the pooled sample of cases and controls rather than an independent reference population; therefore, the inclusion of cases in defining these boundaries may have influenced the observed associations and potentially exaggerated their magnitude. Several limitations pertain to data collection: although lifestyle variables were collected using a structured CRF, because the ultimate source is participant self-report, the risk of recall bias (e.g., for water consumption) is not entirely eliminated for certain variables; the absence of laboratory data such as serum calcium, uric acid, parathyroid hormone, or urinary electrolytes precludes direct testing of the mechanistic interpretations discussed above (particularly the WHR–serum calcium pathway); in addition, some categorical CRF variables (e.g., history of bowel surgery) were defined without distinguishing subtypes. On the analysis side, the model was evaluated using a single 80–20% training-test split, without k-fold cross-validation or external validation; because no SHAP analysis was performed, it could not be determined which variable group the classifier’s performance was primarily attributable to, and, owing to the mathematical identity of BRI and WtHR, these two indices should not be regarded as independent evidence (see above). The cohort overlap of the case group has been transparently addressed above. The contribution of the autoencoder step itself was not tested, because the classifiers were not also fitted to the raw variables for comparison. Finally, the discriminative power of all indices remained weak (AUC < 0.60), which limits their clinical use on their own.

## 5. Conclusions

The findings obtained in the study indicate that anthropometric measurements, clinical data, and lifestyle-related data provide varying degrees of discriminative power between individuals with and without stone disease, depending on the analysis method employed. While the classical BMI carried no discriminative value (AUC = 0.498, 95% CI: 0.465–0.531), the five next-generation indices reflecting central adiposity (ABSI, BRI, WtHR, ConI, WHR) showed higher but weak discriminative power (AUC 0.547–0.564). The hybrid AI model, jointly evaluating anthropometric, clinical, and lifestyle data, achieved 77% accuracy with XGBoost in distinguishing patients with and without stones, suggesting that a multivariable approach may be more valuable than the modest discriminative power of the individual indices alone. These findings are a hypothesis-generating risk-stratification signal and do not support the use of these indices as a screening tool. Validation in independent cohorts is required before clinical implementation.

## Figures and Tables

**Table 1:** Formulas for the anthropometric indices.

Index	Formula
BMI	Body Weight (kg)/Height^2^ (m)
ABSI	Waist Circumference (m)/[BMI2/3 (kg/m^2^) × Height1/2 (m)]
BRI	364.2 − 365.5 × √[1 − ((Waist Circumference (m)/2π)/(0.5 × Height (m)))^2^]
WtHR	Waist Circumference (cm)/Height (cm)
ConI	Waist Circumference (m)/[0.109 × √(Body Weight (kg)/Height (m))]
WHR	Waist Circumference (cm)/Hip Circumference (cm)

BMI: Body Mass Index; ABSI: A Body Shape Index; BRI: Body Roundness Index; WtHR: Waist-to-Height Ratio; ConI: Conicity Index; WHR: Waist-to-Hip Ratio.

**Table 2:** Hyperparameters of the models.

Model	Hyperparameter	Value
XGBoost	Number of estimators	100
	Maximum depth	6
	Learning rate	0.3
Cubic SVM	Kernel	Polynomial
	Degree	3
	C	1.0
Fine KNN	Number of neighbors	1
	Distance metric	Euclidean
Logistic Regression	Maximum iterations	1000
	Regularization	L2
	C	1.0
Autoencoder	Encoder dimension	8
	Activation function	ReLU
	Optimizer	Adam
	Loss function	MSE
	Epochs	200
	Batch size	64

**Table 3:** Demographic, clinical, and anthropometric characteristics of the participants (stone vs. no stone comparison).

Variable	No Stone (*n* = 633)	Stone (*n* = 562)	*p*
Part A—Basic anthropometric measurements			
Age (years)	51.0 (36.0–64.0)	47.5 (38.0–60.0)	0.047
Height (cm)	171.0 (165.0–177.0)	170.0 (164.0–175.0)	<0.001
Weight (kg)	78.0 (70.0–86.0)	77.0 (68.0–85.0)	0.025
Waist circumference (cm)	94.0 (87.0–102.0)	96.0 (88.0–103.0)	0.057
Hip circumference (cm)	102.0 (96.0–110.0)	101.0 (95.0–108.0)	0.154
Part B—Formulated anthropometric indices			
BMI (kg/m^2^)	26.22 (24.21–29.32)	26.57 (23.99–29.34)	0.919
ABSI	0.0806 (0.0763–0.0846)	0.0814 (0.0777–0.0868)	<0.001
BRI	4.34 (3.36–5.50)	4.74 (3.68–5.73)	<0.001
WtHR	0.55 (0.50–0.60)	0.57 (0.52–0.61)	<0.001
ConI	1.28 (1.21–1.35)	1.29 (1.24–1.38)	<0.001
WHR	0.91 (0.86–0.96)	0.92 (0.88–0.97)	0.005
Part C—Clinical, demographic, and lifestyle characteristics			
Sex—Female	189 (29.9%)	191 (34.0%)	0.142
Education level			0.071
Literate	31 (4.9%)	26 (4.6%)	
Primary Education	214 (33.8%)	173 (30.8%)	
High School	220 (34.8%)	231 (41.1%)	
Bachelor’s Degree	142 (22.4%)	121 (21.5%)	
Master’s Degree	26 (4.1%)	11 (2.0%)	
Diabetes—Yes	83 (13.1%)	77 (13.7%)	0.831
Hypertension—Yes	165 (26.1%)	101 (18.0%)	0.001
Cardiovascular disease—Yes	82 (13.0%)	54 (9.6%)	0.084
Inflammatory bowel disease—Yes	4 (0.6%)	7 (1.2%)	0.421
History of bowel surgery—Yes	27 (4.3%)	9 (1.6%)	0.012
Family history of stone disease—Yes	149 (23.5%)	285 (50.7%)	<0.001
Daily water consumption (L)	2.00 (1.50–2.00)	2.00 (1.50–2.00)	0.725
Daily physical activity level			0.563
Low	104 (16.4%)	80 (14.2%)	
Routine Daily Activities	458 (72.4%)	415 (73.8%)	
Regular Exercise	71 (11.2%)	67 (11.9%)	
Tea consumption			0.725
None	21 (3.3%)	16 (2.8%)	
1–2 cups/day	176 (27.8%)	142 (25.3%)	
4–5 cups/day	190 (30.0%)	176 (31.3%)	
>5 cups/day	246 (38.9%)	228 (40.6%)	
Coffee consumption			0.038
None	304 (48.0%)	244 (43.4%)	
1–2 cups/day	289 (45.7%)	269 (47.9%)	
4–5 cups/day	29 (4.6%)	44 (7.8%)	
>5 cups/day	11 (1.7%)	5 (0.9%)	
Salt intake			0.455
None	57 (9.0%)	37 (6.6%)	
Low	128 (20.2%)	122 (21.7%)	
Normal	397 (62.7%)	359 (63.9%)	
High	51 (8.1%)	44 (7.8%)	
Animal protein intake			0.005
Low	89 (14.1%)	87 (15.5%)	
Normal	384 (60.7%)	290 (51.6%)	
High	160 (25.3%)	185 (32.9%)	
Climate type			<0.001
Mild	342 (54.0%)	235 (41.8%)	
Cold	273 (43.1%)	290 (51.6%)	
Hot	18 (2.8%)	37 (6.6%)	
Occupation type—Sedentary	218 (34.4%)	205 (36.5%)	0.500
Medication associated with stone formation—Yes	22 (3.5%)	59 (10.5%)	<0.001
Sweating			<0.001
None	228 (36.0%)	140 (24.9%)	
Little	261 (41.2%)	289 (51.4%)	
High	144 (22.7%)	133 (23.7%)	
Smoking—Yes	242 (38.2%)	213 (37.9%)	0.954
Alcohol—Yes	71 (11.2%)	73 (13.0%)	0.395

The normality of continuous variables was assessed using the Shapiro–Wilk test; as all continuous variables were non-normally distributed, they are presented as median (interquartile range, IQR), and between-group comparisons were performed using the Mann–Whitney U test. Categorical variables are presented as *n* (%), and between-group comparisons were performed using the chi-square test.

**Table 4:** Performance comparison of classifiers for stone vs. no stone classification.

Metric	XGBoost	Cubic SVM	Fine KNN	Logistic Regression
Accuracy (%)	77.0	68.2	70.7	69.0
Macro Precision (%)	76.8	67.8	70.6	68.6
Macro Recall (%)	77.1	67.4	70.8	68.5
Macro F1-Score (%)	76.9	67.5	70.6	68.5
MCC	0.54	0.35	0.41	0.37
Cohen’s Kappa	0.54	0.35	0.41	0.37
Sensitivity (%)	78.3	60.4	71.7	63.2
Specificity (%)	75.9	74.4	69.9	73.7
PPV (%)	72.2	65.3	65.5	65.7
NPV (%)	81.5	70.2	75.6	71.5

Sensitivity, specificity, PPV, and NPV refer to the stone-present class.

**Table 5:** Association of anthropometric indices with urinary system stone disease: quartile-based multivariable logistic regression analysis.

Index/Quartile	Range	Model 1 aOR (95% CI)	*p*	Model 2 aOR (95% CI)	*p*
BMI					
Q1	16.53–24.03	1.00 (ref)		1.00 (ref)	
Q2	24.09–26.37	0.74 (0.53–1.03)	0.074	0.73 (0.51–1.03)	0.075
Q3	26.4–29.32	1.05 (0.76–1.45)	0.765	1.02 (0.72–1.45)	0.894
Q4	29.32–44.08	0.94 (0.68–1.30)	0.718	0.89 (0.63–1.27)	0.523
P for trend		0.736		0.999	
ABSI					
Q1	0.04964–0.0771	1.00 (ref)		1.00 (ref)	
Q2	0.07711–0.08105	1.34 (0.96–1.87)	0.081	1.54 (1.08–2.21)	0.017
Q3	0.08105–0.08568	1.13 (0.81–1.57)	0.484	1.37 (0.95–1.96)	0.088
Q4	0.08576–0.1184	2.20 (1.57–3.08)	<0.001	2.57 (1.78–3.70)	<0.001
P for trend		<0.001		<0.001	
BRI					
Q1	0.9059–3.521	1.00 (ref)		1.00 (ref)	
Q2	3.54–4.529	1.21 (0.87–1.68)	0.257	1.44 (1.01–2.06)	0.047
Q3	4.533–5.658	1.87 (1.34–2.60)	<0.001	2.29 (1.59–3.30)	<0.001
Q4	5.66–12.44	1.70 (1.22–2.37)	0.002	1.90 (1.32–2.74)	<0.001
P for trend		<0.001		<0.001	
WtHR					
Q1	0.3446–0.5086	1.00 (ref)		1.00 (ref)	
Q2	0.5096–0.5588	1.21 (0.87–1.68)	0.257	1.44 (1.01–2.06)	0.047
Q3	0.559–0.6101	1.87 (1.34–2.60)	<0.001	2.29 (1.59–3.30)	<0.001
Q4	0.6102–0.8533	1.70 (1.22–2.37)	0.002	1.90 (1.32–2.74)	<0.001
P for trend		<0.001		<0.001	
ConI					
Q1	0.7982–1.222	1.00 (ref)		1.00 (ref)	
Q2	1.222–1.285	1.53 (1.09–2.13)	0.013	1.76 (1.22–2.55)	0.003
Q3	1.285–1.365	1.87 (1.34–2.62)	<0.001	2.27 (1.58–3.26)	<0.001
Q4	1.365–1.791	2.17 (1.55–3.05)	<0.001	2.62 (1.80–3.81)	<0.001
P for trend		<0.001		<0.001	
WHR					
Q1	0.4636–0.8716	1.00 (ref)		1.00 (ref)	
Q2	0.8718–0.9149	1.39 (0.99–1.94)	0.054	1.58 (1.10–2.27)	0.013
Q3	0.9151–0.9674	1.45 (1.03–2.02)	0.032	1.72 (1.20–2.48)	0.003
Q4	0.9677–1.282	1.85 (1.30–2.62)	<0.001	1.95 (1.32–2.87)	<0.001
P for trend		<0.001		<0.001	

Model 1: adjusted for age and sex. Model 2 (fully adjusted model): additionally adjusted, beyond Model 1, for education level, hypertension, diabetes, cardiovascular disease, smoking, alcohol use, family history of stone disease, daily water consumption, daily physical activity level, climate type, and medication use associated with stone formation. Quartiles (Q1–Q4) were derived from the distribution of each index across the entire sample (*n* = 1195), with the lowest quartile (Q1) used as the reference category. aOR: adjusted odds ratio; CI: confidence interval. Because BRI and WtHR are monotonic transformations of one another by formulation (Spearman ρ = 1.000), they yielded identical results.

**Table 6:** Comparison of the discriminative power of BMI and next-generation anthropometric indices by ROC curve analysis.

Index	AUC	95% CI
BMI	0.498	0.465–0.531
ABSI	0.564	0.531–0.596
BRI	0.556	0.524–0.589
WtHR	0.556	0.524–0.589
ConI	0.564	0.532–0.597
WHR	0.547	0.515–0.580

95% confidence intervals for the AUC values were calculated using the Hanley–McNeil method. Because BRI and WtHR are mathematically monotonic transformations of one another, they yielded identical AUC values.

## Data Availability

The data presented in this study are available on request from the corresponding author due to privacy and ethical restrictions related to patient data.

## Supplementary Materials

The following supporting information can be downloaded at: https://www.mdpi.com/article/10.3390/diagnostics16213557/s1, Table S1: Dataset description; Table S2: Sensitivity analysis: association of anthropometric indices with urinary system stone disease after adjustment for additional covariates; Table S3: STROBE checklist for case–control studies; Table S4: TRIPOD+AI checklist; Figure S1: Spearman correlation heatmap between the basic anthropometric measurements (Part A) and the derived anthropometric indices (Part B) (*n* = 1195). The correlation between BRI and WtHR is 1.00, owing to the mathematical identity of their formulations. A strong correlation was also observed between ABSI and ConI (ρ = 0.95), consistent with these two indices yielding similar results in the multivariable analyses.

## Abbreviations

The following abbreviations are used in this manuscript:

ABSI	A Body Shape Index
AI	Artificial Intelligence
aOR	Adjusted Odds Ratio
AUC	Area Under the Curve
BMI	Body Mass Index
BRI	Body Roundness Index
CI	Confidence Interval
ConI	Conicity Index
CRF	Case Report Form
FN	False Negative
FP	False Positive
GBD	Global Burden of Disease
IQR	Interquartile Range
KNN	K-Nearest Neighbor
MCC	Matthews Correlation Coefficient
NHANES	National Health and Nutrition Examination Survey
NPV	Negative Predictive Value
PPV	Positive Predictive Value
ReLU	Rectified Linear Unit
ROC	Receiver Operating Characteristic
SD	Standard Deviation
SHAP	SHapley Additive exPlanations
SVM	Support Vector Machine
TN	True Negative
TP	True Positive
WHR	Waist-to-Hip Ratio
WtHR	Waist-to-Height Ratio
XGBoost	Extreme Gradient Boosting
ZJU	Zhejiang University
