# Supplementary material for: Association and Discrimination of Urinary System Stone Disease Using Anthropometric Indices, Lifestyle Behaviors, and Artificial Intelligence: A Multicenter Case–Control Study
Source: Diagnostics (Basel). 2026 Oct 28;16(21):3557. doi: 10.3390/diagnostics16213557 (PMC13762050; doi:10.3390/diagnostics16213557)
Supplement: Supplementary file 1 [file diagnostics-16-03557-s001.zip › diagnostics-4609214-supplementary.pdf]

diagnostics

MDPI

Supplementary Materials

Table S1. Dataset description.

Variable	Description	Unit / Coding
	Part A — Basic anthropometric measurements	
A1	Age	years
A2	Height	cm
A3	Weight	kg
A4	Waist circumference	cm
A5	Hip circumference	cm
	Part B — Formulated anthropometric indices	
B1	BMI	BMI
B2	ABSI	ABSI
B3	BRI	BRI
B4	WtHR	WtHR
B5	ConI	ConI
B6	WHR	WHR
	Part C — Clinical, demographic, and lifestyle characteristics	
C1	Sex	1: Male, 2: Female
C2	Education level	1: Literate, 2: Primary Education, 3: High School, 4: Bachelor's Degree, 5: Master's Degree
C3	Diabetes	1: No, 2: Yes
C4	Hypertension	1: No, 2: Yes
C5	Cardiovascular disease	1: No, 2: Yes
C6	Inflammatory bowel disease	1: No, 2: Yes
C7	History of bowel surgery	1: No, 2: Yes
C8	Family history of stone disease	1: No, 2: Yes
C9	Daily water consumption	L/day
C10	Daily physical activity level	0: Low, 1: Routine Daily Activities, 2: Regular Exercise
C11	Tea consumption	0: None, 1: 1–2 cups/day, 2: 4–5 cups/day, 3: >5 cups/day
C12	Coffee consumption	0: None, 1: 1–2 cups/day, 2: 4–5 cups/day, 3: >5 cups/day
C13	Salt intake	0: None, 1: Low, 2: Normal, 3: High
C14	Animal protein intake	0: Low, 1: Normal, 2: High
C15	Climate type	0: Mild, 1: Cold, 2: Hot
C16	Occupation type	1: Active, 2: Sedentary
C17	Medication associated with stone formation	0: No, 1: Yes
C18	Sweating	0: None, 1: Little, 2: High
C19	Smoking	0: No, 1: Yes
C20	Alcohol Consumption	0: No, 1: Yes

BMI: Body Mass Index; ABSI: A Body Shape Index; BRI: Body Roundness Index; WtHR: Waist-to-Height Ratio; ConI: Conicity Index; WHR: Waist-to-Hip Ratio. For index formulas, see Table 1.

Table S2. Sensitivity analysis: association of anthropometric indices with urinary system stone disease after adjustment for additional covariates.

Index / Quartile	Model 2 (primary, as reported in Table 5) aOR (95% CI)	*p*	Model 2 (sensitivity analysis – expanded covariate set) aOR (95% CI)	*p*
BMI				
Q1	1.00 (ref)		1.00 (ref)	
Q2	0.73 (0.51–1.04)	0.079	0.68 (0.48–0.98)	0.039
Q3	1.02 (0.72–1.44)	0.921	0.97 (0.68–1.38)	0.854
Q4	0.91 (0.64–1.30)	0.599	0.85 (0.59–1.23)	0.397
P for trend		*0.937*		*0.827*
ABSI				
Q1	1.00 (ref)		1.00 (ref)	
Q2	1.52 (1.07–2.18)	0.021	1.52 (1.06–2.18)	0.024
Q3	1.34 (0.93–1.92)	0.115	1.36 (0.95–1.97)	0.096
Q4	2.50 (1.73–3.60)	<0.001	2.45 (1.69–3.55)	<0.001
P for trend		*<0.001*		*<0.001*
BRI				
Q1	1.00 (ref)		1.00 (ref)	
Q2	1.45 (1.01–2.08)	0.043	1.45 (1.00–2.09)	0.049
Q3	2.30 (1.59–3.32)	<0.001	2.19 (1.50–3.19)	<0.001
Q4	1.89 (1.31–2.73)	<0.001	1.85 (1.26–2.70)	0.001
P for trend		*<0.001*		*<0.001*
WtHR				
Q1	1.00 (ref)		1.00 (ref)	
Q2	1.45 (1.01–2.08)	0.043	1.45 (1.00–2.09)	0.049
Q3	2.30 (1.59–3.32)	<0.001	2.19 (1.50–3.19)	<0.001
Q4	1.89 (1.31–2.73)	<0.001	1.85 (1.26–2.70)	0.001
P for trend		*<0.001*		*<0.001*
ConI				
Q1	1.00 (ref)		1.00 (ref)	
Q2	1.79 (1.24–2.58)	0.002	1.77 (1.22–2.58)	0.003
Q3	2.21 (1.54–3.18)	<0.001	2.26 (1.56–3.27)	<0.001
Q4	2.57 (1.77–3.74)	<0.001	2.51 (1.71–3.68)	<0.001
P for trend		*<0.001*		*<0.001*
WHR				
Q1	1.00 (ref)		1.00 (ref)	
Q2	1.58 (1.09–2.27)	0.014	1.56 (1.08–2.25)	0.018
Q3	1.78 (1.23–2.56)	0.002	1.69 (1.17–2.46)	0.005
Q4	1.92 (1.30–2.84)	0.001	1.82 (1.23–2.70)	0.003
P for trend		*<0.001*		*0.003*

The primary Model 2 (fully adjusted model), reported in Table 5, was adjusted for age, sex, education level, hypertension, diabetes, cardiovascular disease, smoking, alcohol use, family history of stone disease, daily water consumption, daily physical activity level, climate type, and medication use associated with stone formation. As a sensitivity analysis, this model was re-estimated after additionally adjusting for four covariates that showed statistically significant univariate differences between the stone and stone-free groups in Table 3 but were not included in the primary specification: history of bowel surgery, animal protein intake, coffee consumption, and sweating (the latter three entered as categorical variables, consistent with the treatment of education level, daily physical activity level, and climate type in the primary model). Quartiles (Q1–Q4) were derived from the distribution of each index across the entire sample (*n*=1195), with Q1 used as the reference category,

consistent with the primary analysis. aOR: adjusted odds ratio; CI: confidence interval. Interpretation: The pattern of associations observed in the primary analysis remained materially unchanged after adjustment for the additional covariates. BMI showed no significant association in any quartile under either specification (p-trend=0.937 vs. 0.827). All five next-generation anthropometric indices (ABSI, BRI, WtHR, ConI, and WHR) retained statistically significant positive trends across quartiles in the sensitivity analysis (all p-trend≤0.003), with only minor attenuation of the point estimates. These findings indicate that the primary results are robust to this expanded adjustment set.

Table S3. STROBE checklist for case–control studies.

	Item No	Recommendation	Location in manuscript (section)
**Title and abstract**	1	(a) Indicate the study's design with a commonly used term in the title or the abstract	Title ("A Multicenter Case-Control Study"); Abstract (Methods)
		(b) Provide in the abstract an informative and balanced summary of what was done and what was found	Abstract (Background/Objectives, Methods, Results, Conclusions)
		**Introduction**	
Background/rationale	2	Explain the scientific background and rationale for the investigation being reported	1. Introduction (paragraphs 1-5)
Objectives	3	State specific objectives, including any prespecified hypotheses	1. Introduction (final paragraph: hypothesis and two aims)
		**Methods**	
Study design	4	Present key elements of study design early in the paper	2.1 Study Design and Setting; Abstract (Methods)
Setting	5	Describe the setting, locations, and relevant dates, including periods of recruitment, exposure, follow-up, and data collection	2.1 Study Design and Setting (six tertiary care centers in Turkey, January 2025 to June 2025); 2.3 Data Collection. Follow-up: not applicable.
Participants	6	(a) Give the eligibility criteria, and the sources and methods of case ascertainment and control selection. Give the rationale for the choice of cases and controls	2.2 Participants (case definition, source of controls, exclusion criteria). A separate rationale for the choice of cases and controls is not reported.
		(b) For matched studies, give matching criteria and the number of controls per case	Not applicable (cases and controls were not matched).
Variables	7	Clearly define all outcomes, exposures, predictors, potential confounders, and effect modifiers. Give diagnostic criteria, if applicable	2.2 Participants (outcome and diagnostic criteria); 2.3 Data Collection and 2.4 Anthropometric Measurements (variables, Table 1); 2.5 Variable Grouping; 2.7 Statistical Analysis (covariates of Models 1 and 2); Table S1.
Data sources/ measurement	8*	For each variable of interest, give sources of data and details of methods of assessment (measurement). Describe comparability of assessment methods if there is more than one group	2.3 Data Collection (structured case report form); 2.4 Anthropometric Measurements (measurement of waist and hip circumference by trained nurses with a non-stretchable tape; formulas in Table 1). The same form and measurement protocol were applied at all six centers.

	Item No	Recommendation	Location in manuscript (section)
Bias	9	Describe any efforts to address potential sources of bias	2.2 Participants (outcome confirmed by imaging, surgical and clinical history; exclusion of single kidney and chronic kidney disease); 2.3 and 2.4 (structured form, trained nurses); 2.6 (autoencoders fitted on training data only); 2.7 (Model 1 and Model 2 adjustment, sensitivity analysis); Limitations (recall bias, pooled-sample quartiles). Bias from control selection is not discussed separately.
Study size	10	Explain how the study size was arrived at	Not reported. No sample size calculation is described.
Quantitative variables	11	Explain how quantitative variables were handled in the analyses. If applicable, describe which groupings were chosen and why	2.7 Statistical Analysis (each index divided into quartiles of the whole sample, trend test on quartile rank; education, physical activity and climate entered as dummy variables); Limitations (pooled-sample quartiles).
Statistical methods	12	(a) Describe all statistical methods, including those used to control for confounding	2.7 Statistical Analysis (group comparisons, logistic regression Models 1 and 2, trend test, ROC/AUC); 2.6 (classification models and performance metrics).
		(b) Describe any methods used to examine subgroups and interactions	Not applicable (no subgroup or interaction analyses are reported).
		(c) Explain how missing data were addressed	2.3 Data Collection (no missing values in any of the 31 variables; no imputation needed).
		(d) If applicable, explain how matching of cases and controls was addressed	Not applicable (no matching; see item 6b).
		(e) Describe any sensitivity analyses	2.7 Statistical Analysis (Model 2 re-estimated with four additional covariates); 3. Results (sensitivity analysis); Table S2.
		**Results**	
Participants	13*	(a) Report numbers of individuals at each stage of study—eg numbers potentially eligible, examined for eligibility, confirmed eligible, included in the study, completing follow-up, and analysed	3. Results (first paragraph); Figure 1 (562 cases, 633 controls, 1195 analysed); 2.2 Participants. The numbers screened for eligibility and the numbers excluded are not reported.
		(b) Give reasons for non-participation at each stage	Not reported (2.2 lists the exclusion criteria; numbers excluded and reasons for non-participation are not given).
		(c) Consider use of a flow diagram	Figure 1
Descriptive data	14*	(a) Give characteristics of study participants (eg demographic, clinical, social) and information on exposures and potential confounders	3. Results (first paragraphs); Table 3 (all characteristics by stone status).
		(b) Indicate number of participants with missing data for each variable of interest	2.3 Data Collection (no missing data for any variable).

	Item No	Recommendation	Location in manuscript (section)
Outcome data	15*	Report numbers in each exposure category, or summary measures of exposure	Table 3 (median and IQR of each index in cases and controls); Table 5 (quartile boundaries). The numbers of cases and controls in each quartile are not reported.
Main results	16	(a) Give unadjusted estimates and, if applicable, confounder-adjusted estimates and their precision (eg, 95% confidence interval). Make clear which confounders were adjusted for and why they were included	Table 5 (Model 1 and Model 2 adjusted odds ratios with 95% CI; covariates listed in 2.7 and in the Table 5 footnote); Table 6 (AUC with 95% CI). Unadjusted group comparisons are in Table 3; unadjusted odds ratios are not reported.
		(b) Report category boundaries when continuous variables were categorized	Table 5 (range column with quartile boundaries); 2.7 Statistical Analysis.
		(c) If relevant, consider translating estimates of relative risk into absolute risk for a meaningful time period	Not applicable (the case-control design does not allow estimation of absolute risk; see Limitations).
Other analyses	17	Report other analyses done—eg analyses of subgroups and interactions, and sensitivity analyses	Table S2 (sensitivity analysis); Figure S1 (Spearman correlations, cited in 4. Discussion); Table 6 and Figure 4 (ROC analysis); Table 4 and Figures 2-3 (classifiers).
		**Discussion**	
Key results	18	Summarise key results with reference to study objectives	4. Discussion (first paragraph); Abstract (Results); 5. Conclusions.
Limitations	19	Discuss limitations of the study, taking into account sources of potential bias or imprecision. Discuss both direction and magnitude of any potential bias	Limitations (end of 4. Discussion). The direction of the possible bias from pooled-sample quartiles is stated (associations possibly exaggerated); its magnitude is not quantified.
Interpretation	20	Give a cautious overall interpretation of results considering objectives, limitations, multiplicity of analyses, results from similar studies, and other relevant evidence	4. Discussion (comparison with earlier studies and interpretation); Limitations; 5. Conclusions. Multiplicity of analyses is not discussed.
Generalisability	21	Discuss the generalisability (external validity) of the study results	4. Discussion (multicenter design in six centers; comparison with the population-based NHANES samples); Limitations (no external validation); 5. Conclusions (validation in independent cohorts required). There is no dedicated generalisability paragraph.
		**Other information**	
Funding	22	Give the source of funding and the role of the funders for the present study and, if applicable, for the original study on which the present article is based	Funding statement ("This research received no external funding").

*Give information separately for cases and controls.

Table S4. TRIPOD+AI checklist.

Section/Topic	Item	Development / evaluation[1]	Checklist item	Location in manuscript (section)
			TITLE	
Title	1	D;E	Identify the study as developing or evaluating the performance of a multivariable prediction model, the target population, and the outcome to be predicted	Title; Abstract. The title names the design, the AI approach and the outcome (urinary system stone disease). It does not use the term "prediction model", because the study is framed as association and discrimination.
			ABSTRACT	
Abstract	2	D;E	See TRIPOD+AI for Abstracts checklist	Abstract (structured abstract). The separate TRIPOD+AI for Abstracts checklist was not completed.
			INTRODUCTION	
Background	3a	D;E	Explain the healthcare context (including whether diagnostic or prognostic) and rationale for developing or evaluating the prediction model, including references to existing models	1. Introduction (paragraphs 1-5; existing AI models for stone disease in paragraphs 4 and 5). Whether the model is diagnostic or prognostic is not stated; it separates participants with and without stone disease in a case-control sample.
	3b	D;E	Describe the target population and the intended purpose of the prediction model in the context of the care pathway, including its intended users (e.g., healthcare professionals, patients, public)	Not reported. No care pathway or intended users are defined. Abstract and 5. Conclusions state that the findings are hypothesis-generating and do not support clinical screening.
	3c	D;E	Describe any known health inequalities between sociodemographic groups	Not reported.
Objectives	4	D;E	Specify the study objectives, including whether the study describes the development or validation of a prediction model (or both)	1. Introduction (final paragraph: hypothesis and two aims; the second aim is the development of the hybrid autoencoder-based model and the comparison of four classifiers). Development only; no external validation.
			METHODS	
Data	5a	D;E	Describe the sources of data separately for the development and evaluation datasets (e.g., randomised trial, cohort, routine care or registry data), the rationale for using these data, and representativeness of the data	2.2 Participants (cases and controls recruited at the same six centers); 2.3 Data Collection. No separate evaluation dataset; the test set is a 20% split of the same sample (2.6). Representativeness: 4. Discussion and Limitations.
	5b	D;E	Specify the dates of the collected participant data, including start and end of participant accrual; and, if applicable, end of follow-up	2.1 Study Design and Setting (January 2025 to June 2025). Follow-up: not applicable.
Participants	6a	D;E	Specify key elements of the study setting (e.g., primary care, secondary care, general population) including the number and location of centres	2.1 Study Design and Setting (The individual centers and per-center sample sizes are not given in the text).

	6b	D;E	Describe the eligibility criteria for study participants	2.2 Participants (case definition; exclusion of single kidney and chronic kidney disease).
	6c	D;E	Give details of any treatments received, and how they were handled during model development or evaluation, if relevant	Not reported. Treatments for stone disease are not described; "medication associated with stone formation" was one of the model variables (2.3, Table S1).
Data preparation	7	D;E	Describe any data pre-processing and quality checking, including whether this was similar across relevant sociodemographic groups	2.3 Data Collection (structured case report form; no missing values, no imputation); 2.5 Variable Grouping; 2.6 (autoencoders fitted on the training subset only, test subset only transformed); Table S1 (coding scheme). Scaling or other input transformation is not described.
Outcome	8a	D;E	Clearly define the outcome that is being predicted and the time horizon, including how and when assessed, the rationale for choosing this outcome, and whether the method of outcome assessment is consistent across sociodemographic groups	2.2 Participants (stone status confirmed by imaging findings, surgical history and clinical history). No time horizon (case-control design). Consistency across sociodemographic groups is not reported.
	8b	D;E	If outcome assessment requires subjective interpretation, describe the qualifications and demographic characteristics of the outcome assessors	Not reported.
	8c	D;E	Report any actions to blind assessment of the outcome to be predicted	Not reported.
Predictors	9a	D	Describe the choice of initial predictors (e.g., literature, previous models, all available predictors) and any pre-selection of predictors before model building	1. Introduction (literature on anthropometric indices); 2.3 Data Collection and 2.5 Variable Grouping (all 31 variables used; Table S1). No pre-selection is described.
	9b	D;E	Clearly define all predictors, including how and when they were measured (and any actions to blind assessment of predictors for the outcome and other predictors)	2.3 Data Collection; 2.4 Anthropometric Measurements (measurement protocol, Table 1); Table S1 (definitions and coding). Blinding of predictor assessment is not reported.
	9c	D;E	If predictor measurement requires subjective interpretation, describe the qualifications and demographic characteristics of the predictor assessors	2.4 Anthropometric Measurements (measurements by experienced, trained nurses). Demographic characteristics of the assessors are not reported.
Sample size	10	D;E	Explain how the study size was arrived at (separately for development and evaluation), and justify that the study size was sufficient to answer the research question. Include details of any sample size calculation	Not reported. No sample size calculation is described (2.2 Participants). Total n=1195; test set n=239 (2.6 and 3. Results).
Missing data	11	D;E	Describe how missing data were handled. Provide reasons for omitting any data	2.3 Data Collection (no missing values in any of the 31 variables; no imputation needed).
Analytical methods	12a	D	Describe how the data were used (e.g., for development and evaluation of model performance) in the analysis, including	2.6 Artificial Intelligence Model Development (random 80% training and 20% test split with a fixed random state; autoencoders fitted on the training subset only;

			whether the data were partitioned, considering any sample size requirements	test set of 106 stone and 133 stone-free participants). Sample size requirements for the split are not considered.
	12b	D	Depending on the type of model, describe how predictors were handled in the analyses (functional form, rescaling, transformation, or any standardisation).	2.5 Variable Grouping (three variable subgroups); 2.6 (each subgroup encoded by a separate autoencoder, encoded features concatenated); Table S1 (coding). Rescaling and standardisation are not described.
	12c	D	Specify the type of model, rationale[2], all model-building steps, including any hyperparameter tuning, and method for internal validation	2.6 (hybrid autoencoder plus XGBoost, Cubic SVM, Fine KNN and logistic regression); Table 2 (hyperparameters); Figure 2 (model structure). Internal validation: single hold-out test set. Hyperparameter tuning is not described; Table 2 gives the fixed values used.
	12d	D;E	Describe if and how any heterogeneity in estimates of model parameter values and model performance was handled and quantified across clusters (e.g., hospitals, countries). See TRIPOD-Cluster for additional considerations[3]	Not reported. Heterogeneity across the six centers was not examined.
	12e	D;E	Specify all measures and plots used (and their rationale) to evaluate model performance (e.g., discrimination, calibration, clinical utility) and, if relevant, to compare multiple models	2.6 (accuracy, sensitivity, specificity, PPV, NPV, F1-score, macro-averaged metrics, MCC, Cohen's kappa; equations 1-8; Table 4 compares the four classifiers); 2.7 (ROC/AUC with Hanley-McNeil 95% CI for single indices). Calibration and clinical utility were not assessed.
	12f	E	Describe any model updating (e.g., recalibration) arising from the model evaluation, either overall or for particular sociodemographic groups or settings	Not applicable (evaluation-only item).
	12g	E	For model evaluation, describe how the model predictions were calculated (e.g., formula, code, object, application programming interface)	Not applicable (evaluation-only item).
Class imbalance	13	D;E	If class imbalance methods were used, state why and how this was done, and any subsequent methods to recalibrate the model or the model predictions	Not applicable. No class imbalance method was used; 2.6 notes the class distribution (562 stone-positive and 633 stone-negative cases).
Fairness	14	D;E	Describe any approaches that were used to address model fairness and their rationale	Not reported.
Model output	15	D	Specify the output of the prediction model (e.g., probabilities, classification). Provide details and rationale for any classification and how the thresholds were identified	2.6 (classification into stone present / stone absent, positive class defined); 3. Results (Table 4, Figure 3). Classification thresholds and how they were identified are not described.
Training versus evaluation	16	D;E	Identify any differences between the development and evaluation data in healthcare setting, eligibility criteria, outcome, and predictors	Not applicable. There is no separate evaluation dataset; the test set is a random split of the same sample (2.6).

Ethical approval	17	D;E	Name the institutional research board or ethics committee that approved the study and describe the participant-informed consent or the ethics committee waiver of informed consent	2.1 Study Design and Setting; Institutional Review Board Statement; Informed Consent Statement (Scientific Research Ethics Committee of Eskişehir City Hospital, ESH/BAEK 2025/93, 7 January 2025; written informed consent from all participants).
			OPEN SCIENCE	
Funding	18a	D;E	Give the source of funding and the role of the funders for the present study	Funding statement (no external funding).
Conflicts of interest	18b	D;E	Declare any conflicts of interest and financial disclosures for all authors	Conflicts of Interest statement.
Protocol	18c	D;E	Indicate where the study protocol can be accessed or state that a protocol was not prepared	Not reported.
Registration	18d	D;E	Provide registration information for the study, including register name and registration number, or state that the study was not registered	Not reported.
Data sharing	18e	D;E	Provide details of the availability of the study data	Data Availability Statement (available on request from the corresponding author).
Code sharing	18f	D;E	Provide details of the availability of the analytical code[4]	Not reported.
			PATIENT & PUBLIC INVOLVEMENT	
Patient & Public Involvement	19	D;E	Provide details of any patient and public involvement during the design, conduct, reporting, interpretation, or dissemination of the study or state no involvement.	Not reported.
			RESULTS	
Participants	20a	D;E	Describe the flow of participants through the study, including the number of participants with and without the outcome and, if applicable, a summary of the follow-up time. A diagram may be helpful.	3. Results (first paragraph); Figure 1 (562 cases, 633 controls, 1195 analysed). Follow-up: not applicable.
	20b	D;E	Report the characteristics overall and, where applicable, for each data source or setting, including the key dates, key predictors (including demographics), treatments received, sample size, number of outcome events, follow-up time, and amount of missing data. A table may be helpful. Report any differences across key demographic groups.	3. Results and Table 3 (characteristics of cases and controls, including predictors and sex and education); 2.1 (dates); 2.3 (no missing data). Subgroup results by demographic group are not reported.
	20c	E	For model evaluation, show a comparison with the development data of the distribution of important predictors (demographics, predictors, and outcome).	Not applicable (evaluation-only item).
Model development	21	D;E	Specify the number of participants and outcome events in each analysis (e.g., for model development, hyperparameter tuning, model evaluation)	2.6 (random 80/20 split; test set of 106 stone and 133 stone-free participants); 3. Results (test set of 239 participants, Figure 3). The numbers of participants and events in the training set are not stated separately.

Model specification	22	D	Provide details of the full prediction model (e.g., formula, code, object, application programming interface) to allow predictions in new individuals and to enable third-party evaluation and implementation, including any restrictions to access or re-use (e.g., freely available, proprietary)[5]	Not reported. The full model (autoencoder weights, classifier objects, code) is not provided. Architecture and hyperparameters are in 2.6, Table 2 and Figure 2.
Model performance	23a	D;E	Report model performance estimates with confidence intervals, including for any key subgroups (e.g., sociodemographic). Consider plots to aid presentation.	3. Results; Table 4 and Figure 3 (test-set metrics and confusion matrices); Table 6 and Figure 4 (AUC with 95% CI for single indices). Confidence intervals for the classifier metrics and subgroup performance are not reported.
	23b	D;E	If examined, report results of any heterogeneity in model performance across clusters. See TRIPOD Cluster for additional details[3].	Not applicable. Heterogeneity across centers was not examined (see item 12d).
Model updating	24	E	Report the results from any model updating, including the updated model and subsequent performance	Not applicable (evaluation-only item).
			DISCUSSION	
Interpretation	25	D;E	Give an overall interpretation of the main results, including issues of fairness in the context of the objectives and previous studies	4. Discussion (key results, comparison with earlier studies); 5. Conclusions. Fairness is not discussed.
Limitations	26	D;E	Discuss any limitations of the study (such as a non-representative sample, sample size, overfitting, missing data) and their effects on any biases, statistical uncertainty, and generalizability	Limitations (end of 4. Discussion: single 80%-20% split without cross-validation or external validation, no SHAP analysis, autoencoder contribution not tested, self-reported lifestyle data, pooled-sample quartiles, case-control design); 4. Discussion (ratio structure of the indices). Overfitting is not discussed separately.
Usability of the model in the context of current care	27a	D	Describe how poor quality or unavailable input data (e.g., predictor values) should be assessed and handled when implementing the prediction model	Not applicable. Implementation is not proposed (Abstract and 5. Conclusions: the findings do not support clinical screening).
	27b	D	Specify whether users will be required to interact in the handling of the input data or use of the model, and what level of expertise is required of users	Not applicable (see item 27a).
	27c	D;E	Discuss any next steps for future research, with a specific view to applicability and generalizability of the model	5. Conclusions (validation in independent cohorts required before clinical implementation); Limitations (no external validation).

[1] D = items relevant only to the development of a prediction model; E = items relating solely to the evaluation of a prediction model; D;E = items applicable to both the development and evaluation of a prediction model.

[2] Separately for all model building approaches.

[3] TRIPOD-Cluster is a checklist of reporting recommendations for studies developing or validating models that explicitly account for clustering or explore heterogeneity in model

performance (e.g., at different hospitals or centres). Debray et al, BMJ 2023; 380: e071018 [DOI: 10.1136/bmj-2022-071018].

[4] This relates to the analysis code, for example, any data cleaning, feature engineering, model building, evaluation.

[5] This relates to the code to implement the model to get estimates of risk for a new individual.

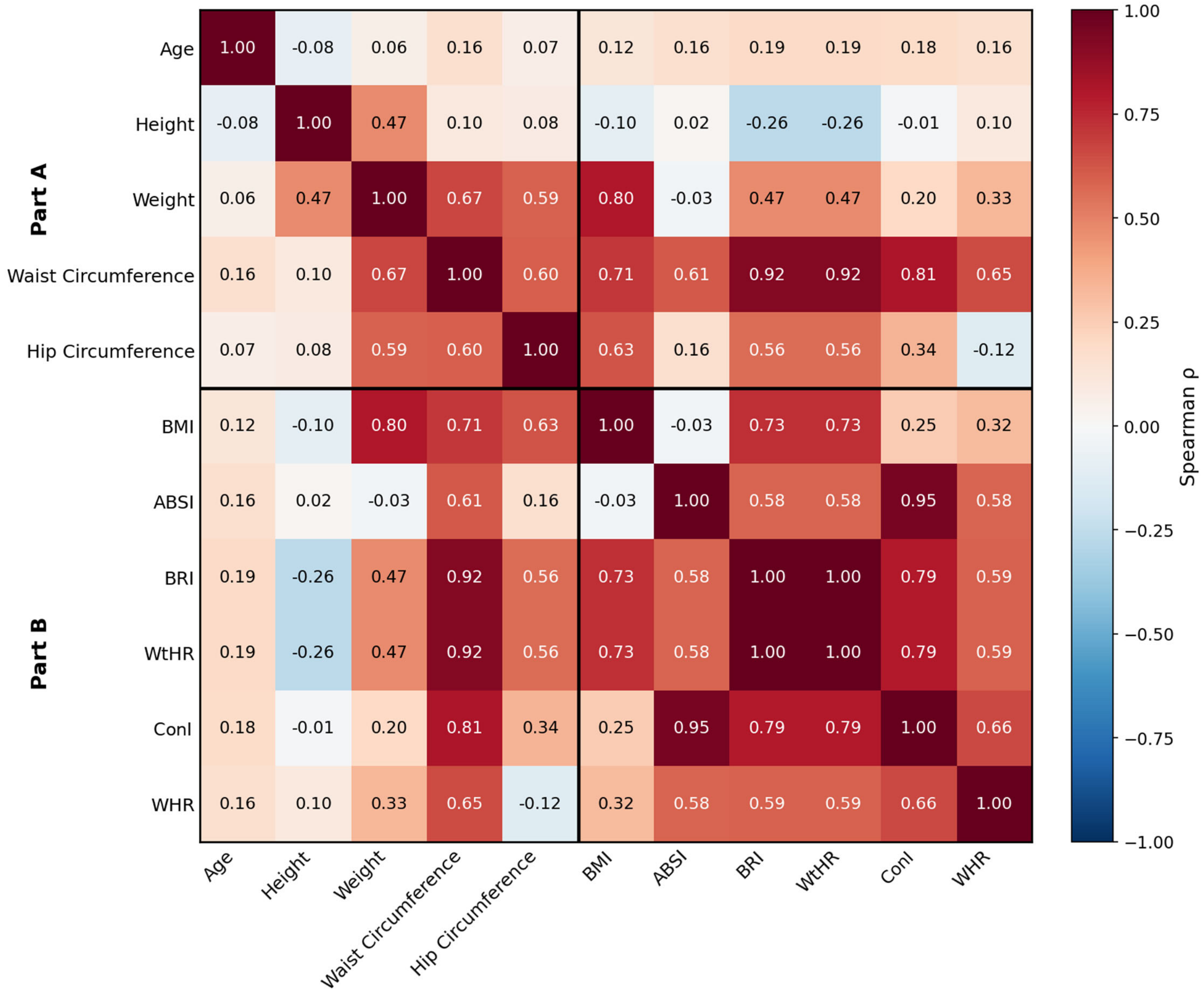

Figure S1. Spearman correlation heatmap between the basic anthropometric measurements (Part A) and the derived anthropometric indices (Part B) (*n*=1195). The correlation between BRI and WtHR is 1.00, owing to the mathematical identity of their formulations. A strong correlation was also observed between ABSI and ConI (ρ=0.95), consistent with these two indices yielding similar results in the multivariable analyses.
